# Role of Uremic Toxins in Early Vascular Ageing and Calcification

**DOI:** 10.3390/toxins13010026

**Published:** 2021-01-03

**Authors:** Nikolaos C. Kyriakidis, Gabriela Cobo, Lu Dai, Bengt Lindholm, Peter Stenvinkel

**Affiliations:** 1 One Health Research Group, Faculty of Medicine, Universidad de Las Américas (UDLA), Antigua Vía a Nayón, Quito EC170124, Ecuador; nikolaos.kyriakidis@udla.edu.ec; 2 Nephrology Department, Hospital de Especialidades Eugenio Espejo, Av. Yahuachi s/n, Quito EC170403, Ecuador; gabriela.cobo@hee.gob.ec; 3 Division of Renal Medicine and Baxter Novum, Department of Clinical Science, Intervention and Technology, Karolinska Institutet, Huddinge, 141 86 Stockholm, Sweden; lu.dai@ki.se (L.D.); bengt.lindholm@ki.se (B.L.)

**Keywords:** chronic kidney disease, uremic toxins, vascular calcification, cardiovascular disease, vascular smooth muscle cells

## Abstract

In patients with advanced chronic kidney disease (CKD), the accumulation of uremic toxins, caused by a combination of decreased excretion secondary to reduced kidney function and increased generation secondary to aberrant expression of metabolite genes, interferes with different biological functions of cells and organs, contributing to a state of chronic inflammation and other adverse biologic effects that may cause tissue damage. Several uremic toxins have been implicated in severe vascular smooth muscle cells (VSMCs) changes and other alterations leading to vascular calcification (VC) and early vascular ageing (EVA). The above mentioned are predominant clinical features of patients with CKD, contributing to their exceptionally high cardiovascular mortality. Herein, we present an update on pathophysiological processes and mediators underlying VC and EVA induced by uremic toxins. Moreover, we discuss their clinical impact, and possible therapeutic targets aiming at preventing or ameliorating the harmful effects of uremic toxins on the vasculature.

## 1. Introduction

Chronic kidney disease (CKD) is an increasingly prevalent condition with an estimated worldwide prevalence of 10–12% and an overwhelmingly high mortality rate projecting CKD to become one of the top five causes of death by 2040 [1].

The progressive loss of kidney function is accompanied by the retention of numerous solutes that would normally be excreted in the urine; in so far these substances have been shown to have a negative impact on the function of organs and systems of the body they are called uremic toxins. Traditionally, uremic toxins are classified based on their physico-chemical characteristics into three categories: free water-soluble low molecular weight solutes, protein-bound solutes, and middle molecules [2]. To date, more than hundred solutes have been described as uremic toxins [3]. Uremic toxins have been implicated in many different pathological pathways that may be responsible for complications contributing to the high mortality observed in patients with advanced CKD [4]. Although the study of the uremic milieu is complex and difficult to investigate in research models due to the interactions and effects of these solutes at different levels and in synergic manners, enormous efforts have been made in recent years to depict the clinical phenotype associated with these solutes. Among the different compounds, large uremic toxins belonging to the middle molecules group that comprise among others pro-inflammatory mediators and other cytokines have been estimated to constitute 23% of the number of uremic retention solutes identified as uremic toxins [5]. These solutes deserve special attention because of the challenges for their elimination using conventional dialysis strategies [6]. However, low molecular weight and protein bound solutes also play an important role in the physiopathology and clinical phenotype produced by the uremic milieu. In this review, we discuss the pathophysiological links between the different uremic toxins, with special emphasis on middle molecules, and their impact on vascular calcification (VC) and early vascular ageing (EVA) in CKD, and potential therapeutic strategies to slow down the appearance of this condition.

## 2. Vascular Calcification and Early Vascular Ageing in CKD Patients

The uremic milieu that accompanies CKD has been linked to a clinical model of premature ageing [7], characterized by low grade chronic inflammation, muscle wasting, osteoporosis and frailty, and an extremely high cardiovascular mortality [8,9] which in end stage kidney disease (ESKD) patients is more than 10 times higher compared to the general population [10]. In fact, CKD has been identified as an independent risk factor for cardiovascular disease (CVD), and according to the Global Burden Disease study, 1.4 million deaths from CVD were attributable to impaired kidney function, representing 7.6% of all deaths due to CVD in 2017 [1].

The complete pathologic pathways leading to this high cardiovascular mortality are not fully elucidated, but the presence of EVA seems to be one of the main culprits [7]. Vascular ageing is characterized by a gradual change of vascular structure manifested with VC, loss of arterial elasticity, reduced arterial compliance and endothelial dysfunction, resulting in increased arterial stiffening. The dissociation between chronological and biological age of large arteries is a typical feature of EVA in uremia.

Observational studies report that VC is present in more than 30% of CKD patients, and with prevalence and severity increasing in more advanced stages of CKD, some studies report a prevalence as high as 74% [11,12]. It is generally thought that VC in CKD involves the calcification of the vascular smooth muscle cell (VSMC) layer, secondary to phenotypic modulation, maladaptation, and cellular death. In CKD patients, time on dialysis (increased “dialysis vintage”), calcium-phosphate disorders, diabetes, ageing, and inflammation have been described as risk factors [13].

## 3. Uremic Toxin Mediated Pathways Leading to Vascular Calcification

**Figure 1:**
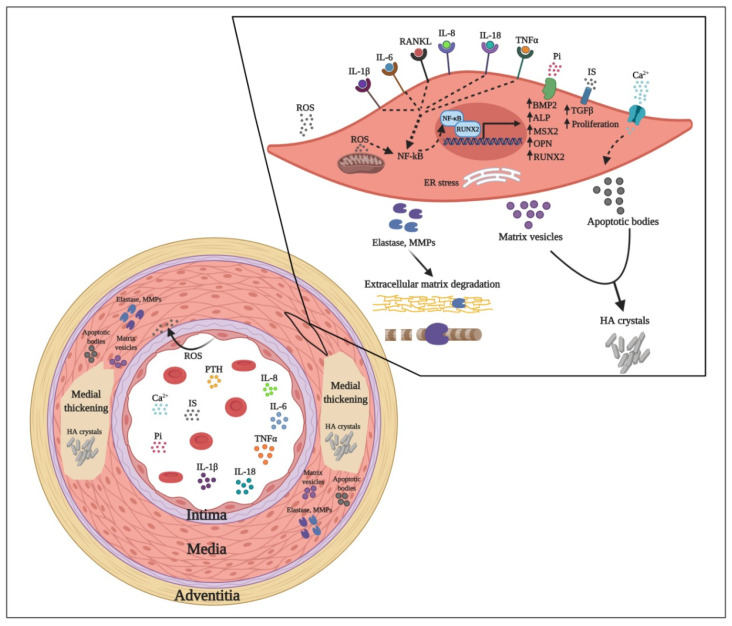
Molecules and pathways leading to medial vascular calcification in CKD. Deteriorating renal function induced by progressing CKD leads to the accumulation of various uremic toxins including Pi, IL-1β, IL-6, TNFα and disruption of Ca^2+^ homeostasis through the upregulation of PTH and FGF-23. Elevated Ca^2+^ levels induce secretion of apoptotic bodies that contribute to hydroxyapatite (HA) crystal formation. Moreover, Pi induces the upregulation of several osteoblast-like transition molecules like BMP2, MSX2 and OPN that initiate the pro-calcifying trans-differentiation of VSMCs. IS stimulates TGFβ expression and medial layer hyperplasia. Uremic toxins such as Pi, IS or pro-inflammatory cytokines acting on endothelial cells induce vasoconstriction, upregulation of extracellular matrix degradation molecules MMP-2 and -9 and oxidative stress. ROS produced by dysregulated endothelial cells and mitochondria of activated VSMCs and cytokine recognition signaling collectively activate NF-kB liberation from its inhibitor and subsequent translocation to the nucleus where it promotes the expression of several pro-inflammatory, pro-apoptotic genes and extracellular matrix degradation molecules such as ALP, elastase and MMP-2 and -9. Ca^2+^ and Pi deposition in the form of HA crystals induces medial VC. Abbreviations: CKD: chronic kidney disease; FGF-23: fibroblast growth factor-23; PTH: parathyroid hormone; VC: vascular calcification; MMPs: matrix metalloproteinases; HA: hydroxyapatite crystal; IS: indoxyl-sulfate; VSMCs: vascular smooth muscle cell; Pi: inorganic phosphate; Ca^2+^: calcium ions; IL: interleukin; TNFα: tumor necrosis factor alpha; TGFβ: transforming growth factor beta; RANKL: receptor activator of nuclear factor kappa-Β ligand; ROS: reactive oxygen species; NF-kB: nuclear factor kappa-Β; RUNX2: Runt-related transcription factor 2; MSX2: Msh homeobox 2 protein; BMP2: bone morphogenetic protein 2; ALP: alkaline phosphatase.

High cardiovascular morbidity and mortality of CKD cases is mainly attributable to medial VC [14], a hallmark of accelerated vascular ageing processes [15,16]. Calcification can occur in both media and intima layers of the vessel wall. However, media calcification is more prevalent in younger CKD patients. Some of the factors and pathways leading to media VC are illustrated in Figure 1.

The observation that calcified lesions present high local expression of the osteoblast-related bone morphogenetic protein 2 (BMP2) and the bone hormone osteocalcin has nourished the hypothesis that VC is an active biological process sharing key common characteristics with classical osteogenesis [17]. Indeed, defective osteogenesis reflected in low bone mineral density and VC (‘vascular ossification’) are intrinsically linked typical features of the uremic phenotype [18].

It is increasingly recognized that VC is a multistep and cell-mediated dynamic process whereby VSMCs progressively adopt an osteoblast-like phenotype with concomitant expression of bone-related genes such as Runt-related transcription factor 2 (RUNX2) also known as core-binding factor subunit alpha-1 (Cbfa1), Msx2 (Msh Homeobox 2), BMP2, and alkaline phosphatase (ALP). Subsequently, trans-differentiated VSMCs begin to express and secrete an array of pro-inflammatory cytokines, extracellular matrix degrading enzymes and matrix vesicles and apoptotic bodies that culminate with the calcification of the media vascular layer [19,20,21].

Though two of the main drivers of VC are hyperphosphatemia and hypercalcemia, a plethora of tightly regulated biological processes are implicated in the pathogenic processes that result in this condition. In particular, various genetic and epigenetic factors, hormonal dysregulation, aberrant mineral homeostasis, induction of pro-inflammatory processes and oxidative stress phenomena (associated with a repressed expression the cytoprotective transcription factor Nuclear factor erythroid 2-related factor 2 (Nrf2) [22], are collectively considered as important contributors to VC processes [23]. A growing body of evidence additionally implicates the gut dysbiosis in increased generation of uremic toxins, thus playing an important role in the bone-vascular axis in CKD [24].

The actual landscape of VC initiation and progression is even more complex, with a variety of cells and soluble mediators forming an interacting network that orchestrates the pro-calcifying processes that give rise to the vascular stiffness. In physiological conditions, VSMCs maintain a certain degree of differentiation plasticity and have a contractile phenotype that confers the contraction and relaxation properties of the blood vessels and maintain vessel wall integrity and structural characteristics. However, under mechanical trauma or certain chemical stimuli, and among those several uremic toxins, VSMCs can switch to a synthetic phenotype that mediates vascular repair and remodeling processes associated with medial hypertrophy and atherosclerosis [25,26].

### 3.1. Mineral Dysregulation

In CKD patients, the deteriorating renal function leads to impaired clearance of phosphate resulting in hyperphosphatemia [27]. As a compensatory mechanism, high phosphate levels trigger fibroblast growth factor-23 (FGF23) synthesis from osteoblasts and osteocytes that, in turn, acts synergistically with Klotho on the cells of the proximal tubule of the kidney, promoting phosphaturia [28]. An adverse effect of CKD that impedes this compensatory mechanism is the downregulation of Klotho observed in CKD; thus, propagating hyperphosphatemia leads to continuous stimulation of FGF23 production. This upregulation of FGF23 is further amplificated by the accumulation of both 1,25-dihydroxyvitamin D [1,25(OH)2D] and parathyroid hormone (PTH) that are secreted as a result of elevated levels of circulating phosphate [27]. High levels of FGF23 are accompanied by an upregulation of the active vitamin-D degradation enzyme 24-hydroxylase (CYP24A1) and concomitant transcriptional suppression of the vitamin-D activating enzyme 1-a-hydroxylase (CYP27B1), thereby suppressing renal synthesis of 1,25(OH)2D [29]. Circulating calcium and phosphate molecules in the presence of fetuin A form primary calciprotein particles (CPPs) of ~9 nm in diameter. These primary CPPs contain amorphous calcium-phosphate and can transform into a larger crystalline phase (>35 nm) named secondary CPPs. While both forms of calcium-phosphate nanoaggregates appear to induce inflammation [30], primary CPPs are more potent inducers of FGF-23 than secondary CPPs [31], further contributing to 1,25(OH)2D downregulation. The ensuing vitamin-D deficiency causes impaired intestinal absorption of phosphorus and calcium that directly stimulate sustained PTH production from parathyroid glands, a condition known as secondary hyperparathyroidism. The feed-forward loop that culminates in PTH overproduction triggers calcium uptake from the bones and its subsequent reabsorption in the kidneys [32]. Excess phosphorus and calcium molecules deposit can further drive VSMCs undergo osteogenesis. This phenotypic switch induces a variety of behavioral changes as trans-differentiated VSMCs adopt the characteristics of synthetic cells that include high elastase secretion, aberrant senescence, migration, apoptosis and induction of extracellular pro-calcifying vesicles release that promote the formation of hydroxyapatite crystals, thus promoting local calcification [33,34,35]. The pro-calcifying vesicles released by VSMCs are of two types: matrix vesicles (30–300 nm in diameter) and apoptotic bodies (50–5000 nm in diameter) [36]. The apoptotic bodies act as a nucleating factor for nascent hydroxyapatite (HA) crystals [37]. The apoptotic death of VSMCs appears to be the accumulative result of mineral metabolism dysregulation, proinflammatory signaling (as for example the overexpression of the well-established uremic toxins, such as, tumor necrosis factor (TNF) and lysosome-mediated decomposition of medium size HA crystals [38,39]. However, in vitro data suggest that also necrotic phenomena contribute to calcified matrix formation [39].

Although these phenomena represent mechanisms whereby CKD-induced mineral dysregulation leads to VC, a more accurate description would be that calcification is the consequence of the aforementioned pro-osteogenic mechanisms combined with the concomitant failure or downregulation of calcification inhibitors, such as vitamin-K dependent matrix-Gla protein (MGP) [40,41], inorganic pyrophosphate (PPi) [42], fetuin A [43], and osteoprotegerin (OPG) [44]. Briefly, MGP binds and inhibits BMP2 and BMP4, thus acting as a calcification inhibitor [45]. Moreover, MGP is found in matrix vesicles preventing their calcification and is an independent predictor of both me9dia and intimal VC [46]. High calcium levels decrease MGP loading in these vesicles, thereby promoting calcification [47,48]. PPi binds directly to the nascent HA crystals inhibiting further accumulation of phosphate [49,50]. Interestingly, PPi can be degraded by ALP, revealing a potential beneficial role for ALP-inhibitor treatments [51]. Fetuin A paradoxically appears to have a VC inhibiting activity through its role in the formation of CPPs in the bloodstream and in matrix vesicles preventing the precipitation of calcium-phosphate in soft tissues [52,53]. The protective role of osteoprotegerin is described later. Alongside phosphate and PTH, additional uremic toxins contribute to VSMCs osteochondrogenic transition and are linked with endothelial cell dysfunction, oxidative stress and excessive inflammation phenomena that are usually strictly interconnected. As Hegner et al. [54] reported that, among 63 uremic retention solutes, a group of middle-sized molecules (interleukin 1 beta (IL-1β), TNF, FGF23 and PTH) stimulated osteoblastic differentiation of mesenchymal stroma cells and VSCMs, these molecules appear as attractive therapeutic targets.

### 3.2. Endothelial Dysfunction

Endothelial cells (ECs) form a monolayer that lines the innermost surface of the blood vessels and display numerous functions such as maintaining vascular integrity, regulating the interchange of molecules and immune cells between blood and underlying tissues, ensuring vascular homeostasis, regulating blood pressure, angiogenesis, and coagulation, and even acting as the perhaps largest endocrine gland of the human body [55]. Though ECs are more relevant in atherosclerotic processes, their central role in regulating the exchange of key molecules that can activate VSMCs as well as the fact that they can be an abundant source of various proinflammatory, proapoptotic, profibrotic cytokines and reactive oxygen species (ROS) highlights their importance in the initiation and propagation of medial VC [56].

Emerging data show that EC dysfunction constitutes one of the earliest steps of the processes that lead to the osteochondrogenic trans-differentiation of VMSCs both during age-related and CKD-induced VC. EC dysfunction is the outcome of several contributory factors such as the accumulation of circulating uremic toxins and pro-inflammatory cytokines that result in an imbalance between oxidative stress and antioxidant mechanisms. Accordingly, chronic exposure of blood vessels to uremic toxins increases NADPH activity and the ensuing ROS overproduction whilst simultaneously downregulating key antioxidative enzymes such as the endothelial nitrous oxide synthases (eNOS) and superoxide dismutases (SODs). Aberrantly produced ROS act on the cells of the underlying medial layer promoting the osteogenic trans differentiation of VSMCs [reviewed in [57]]. An additional effect of uremic toxins on ECs is the induction of matrix metalloproteinase 2 and 9 (MMP-2 and -9) and the downregulation of the tissues inhibitors of metalloproteinases 1 and 2 (TIMP-1 and TIMP-2) that result in increased extracellular matrix degradation [58].

In line with this, in vivo experiments demonstrated that chronic renal failure murine models that presented an accumulation of uremic toxins exhibited a proinflammatory phenotype that led to extensive subendothelial dysfunction, vascular stiffness and cardiac and aortic abnormalities, all hallmarks of CKD, supporting a pivotal role of the vascular uremic milieu on the induction of EC pathology [59].

Phosphate also acts directly on ECs altering their phenotype and provoking vascular dysfunction. In CKD mice, high serum phosphate levels were shown to modulate vascular activity by inducing vasoconstriction and EC detachment [60]. It seems likely that the modulation of EC activity and the pro-apoptotic effect of Pi is mediated by the activation of NADPH and the concomitant inactivation of eNOS that results in reduced nitrous oxide production [61,62]. Moreover, low grade inflammation is recognized as one of the key underlying mechanisms of VC [54]. Central regulators of EC and VSMC phenotype changes are inflammatory and profibrotic cytokines and locally produced ROS. Only a few of these soluble mediators are considered as uremic toxins [3], but recent studies suggest that their inclusion in this group warrants further consideration [5].

### 3.3. Oxidative Stress

Oxidative stress is the pathogenic condition that stems from an imbalance between ROS production and the ROS clearance mechanisms of the organism. Major cellular sources of ROS are mitochondria, NADPH oxidase enzyme (Nox) and 5′-lipoxygenase [63]. The production of ROS consists in the formation of peroxide, superoxide, hydroxyl radical, singlet oxygen and alpha oxygen. Low grade production of ROS occurs intrinsically in cells as they mediate physiological cell processes. Nevertheless, phagocytic cells like monocytes, macrophages and neutrophils can scale up the production of ROS by activating their lysosomal Nox. This process, called respiratory burst, leads to excess ROS production that has both antimicrobial effects but can also cause irreversible DNA damage as ROS oxidize and modify cellular components, preventing them from performing their original functions. Hence, phagocytes have antioxidant enzymes to limit the deleterious effects of ROS metabolizing them to inoffensive chemicals. Important antioxidant enzymes are the superoxide dismutases (SODs), glutathione peroxidase (GPx), catalase (CAT) and peroxiredoxins (PRDX1-6) that reside in mitochondria and the cytoplasm. Hyperactivation of Nox and/or downregulation of antioxidant enzymes generate an oxidative stress.

In the vasculature, ECs and VSMCs have been described as the main sources of ROS that affect homeostasis. Contrary to phagocyte Nox, their endothelial and smooth cell counterparts (Nox 1, Nox 2, Nox 4, and Nox 5) produce basal amounts of ROS that mediate physiologic mechanisms leading to oxidative stress [64]. Additional sources of superoxide anion in the vasculature are eNOS. Oxidative stress is one of the better studied mechanisms that contribute to the uremic phenotype and VC [65,66]. In this sense, increased expression of the Nox subunits p22phox and p47phox and downregulation of the antioxidant enzymes SOD1, SOD2, Gpx1, and PRDX-1 were reported in the aorta of rats with CKD [67]. Other sources of excessive ROS production are bone morphogenetic protein-2 (BMP2) activation of VSMCs Nox that leads to VC [68], calcium-dependent activation of Nox5 in VSMCs [69], inorganic phosphate triggered mitochondrial dysfunction that generates mitochondrial respiratory chain-derived ROS [70,71] and hypoxia-mediated inhibition of the mitochondrial electron transport chain that gives rise to mitochondrial ROS production [72,73,74,75,76]. All these conditions use ROS production as a common intermediate to induce osteoblast/chondrogenic trans-differentiation of VSMCs and VC via the activation of different downstream pathways. More particularly, ROS production from different stimuli can induce the activation and nuclear translocation of several transcription factors, such as NF-kB, hypoxia-inducible factor (HIF)-1α [77], B-catenin, and Msx2, activating transcription factor 4 (ATF4), and CCAAT-enhancer-binding protein homologous protein (CHOP), all of which initiate the transcription program that provokes the VSMC transition from a contractile to a synthetic, pro-calcifying phenotype [66]. The cytoprotective transcription Nrf2 play an important role in protection against oxidative stress and inflammation in CKD [78].

In homeostatic conditions, constitutive Nrf2 expression is low and NRF2 protein is ubiquitinated by Kelch-like ECH-associated protein 1 (KEAP1) marking it for proteasomal degradation. However, uremic toxins and ROS trigger a combination of Nrf2 upregulation and KEAP1 degradation that results in increased nuclear translocation of NRF2 protein and its binding to antioxidant response elements (ARE), thus inducing the expression of cytoprotective genes. Indeed, a genetically suppressed Nrf2 mouse model was found to exhibit accelerated age-related induction of senescence markers and a cerebrovascular pro-inflammatory phenotype [79]. Importantly, activation of the Nrf2-induced cytoprotective genes was found to prevent hyperphosphatemia-triggered VC in kidney VSMSCs by inducing an autophagy process [80].

### 3.4. Pro-Inflammatory Cytokines

The three typical pro-inflammatory cytokines mentioned in most immunological textbooks are TNF, IL-1β, and IL-6 [81], although updated lists include a lot more members of this group, such as IL-8, IL-18, and others. All these five pro-inflammatory cytokines are classified as uremic toxins belonging to the group of middle molecules and their role in VC has been established both from experimental approaches and clinical studies [82]. Pro-inflammatory cytokines are principally expressed by macrophages as a response to infection or tissue damage, although several other leucocytes and non-traditional immune cells have been shown to express different subsets of these cytokines [83]. TNF, IL-1β and IL-6 have several common characteristics and they share several local and systemic effects. They act on local ECs and induce the expression of numerous adhesion molecules (of the selectin and integrin groups of molecules) and they increase the vascular permeability to allow recruited immune cells to access injured tissues and activate recruited leukocytes. On a systemic level, these pro-inflammatory cytokines exert a plethora of actions on different tissues triggering a variety of effects. They are known as endogenous pyrogens acting on the hypothalamus and inducing fever, can be recognized by liver cells inducing the production of the acute phase response reactants and can stimulate the production of different leucocyte subsets from the bone marrow.

#### 3.4.1. Tumor Necrosis Factor

In relation to VC, the effect of TNF is pleiotropic including a direct apoptotic effect of VSMCs as well as downregulation of Klotho, thereby inhibiting excess phosphate discharge [84]. Exposure of TNF on VSMCs induces a panel of osteogenic trans-differentiation markers [85]. In accordance, when embryonic rat VSMCs were stimulated with TNF, an induction of autophagy of these cells was observed that led to their de-differentiation and evoked a switch in their inflammatory, migratory, and proliferative profile [86]. Likewise, in vitro exposure of VSMCs to CPPs resulted in enhanced calcification induced by oxidative stress and elevated TNF release. The direct pro-calcifying effect of TNF was further emphasized by the observation that suppressing TNF expression or blocking TNF receptor type 1 (TNFR1) inhibited further calcification events [30]. Moreover, TNF significantly amplified the pro-calcifying properties of trans-differentiated human VSMC cells [87]. A similar effect was observed in TNF-stimulated human and murine VSMCs that adopted a migratory and proliferative phenotype accompanied by overexpression of MMP-1 and -9 and induction of ROS. Use of (+)-episesamin was found to thwart these changes suggesting that this lignan has therapeutic potential against VC [88]. Additionally, TNF upregulates the expression of classic osteogenic markers, such as RUNX2, osterix, ALP, and bone sialoprotein in a NF-kB-dependent fashion. This sequence of activations promptly triggered matrix mineralization in VSMCs [89]. Stimulation of VSMCs with uremic serum containing elevated levels of TNF and IL-6 induced osteogenic trans-differentiation and calcification of VSMCs that could be counteracted by TNF or IL-6 signaling blockade [90]. Clinical studies reveal a strong link between the progression of coronary artery calcification [91] and carotid artery atherosclerosis [92] and TNF and/or IL-6 serum levels in CKD.

#### 3.4.2. Interleukin-6

IL-6 mediates its functions through binding to the IL-6 receptor (IL-6R) that activates signal-transducing gp130 co-receptor, resulting in Janus kinase/signal transducers and activators of transcription (JAK/STAT) phosphorylation and signaling that culminates with the activation and nuclear translocation of NF-kB, STAT1 and STAT3 transcription factors [93]. Soluble IL-6R (sIL-6R) allows for IL-6 signaling in cells lacking IL-6R as the heterodimer sIL-6R/IL-6 can directly engage membrane-bound gp130. It comes as no surprise that both IL-6 and sIL-6R are independent predictors of mortality in ESKD [5,94]. Indeed, the sIL-6R/IL-6/STAT3 pathway was found to fruitfully evoke human VSMCs trans-differentiation into osteogenic-like cells [95]. It should be emphasized that, besides reduced clearance, increased tissue generation also contributes to increased circulating IL-6 levels in ESKD. It has been estimated that increased tissue generation contribute to about 40% of systemic IL-6 levels in ESKD [5].

Receptor activator of NF-ĸB ligand (RANKL) is a known inducer of VC. In a co-culture system, bone marrow derived macrophages treated with RANKL secreted high levels of IL-6 and TNF and induced VSMC calcification, therefore identifying all three cytokines as key parts of a pro-calcific pathway [96]. Similar results were obtained in a OPG and apolipoprotein E (ApoE) deficient mouse model. OPG is a decoy receptor for RANKL that upon recognition ablates it’s signaling. VSMCs isolated from ApoE−/− OPG−/− mice displayed accelerated osteochondrogenic properties compared to ApoE−/− OPG+/+ murine VSMCs and this pro-calcific phenotype was mediated by IL-6 [97]. Human recombinant IL-6 stimulated human umbilical artery VSMCs obtained in an osteogenic phenotype and initiated extracellular calcification processes through upregulation of the osteogenic differentiation factor BMP2 [98]. The synergistic pro-calcific effect of high phosphate and IL-6 levels on VSMCs was shown to be reverted by the anti-ageing agent resveratrol [99]. Several multicenter clinical studies including CKD patients of different ethnicities have demonstrated that elevated serum IL-6 is an independent risk factor for VC progression and mortality [91,92,100,101,102,103,104,105,106].

#### 3.4.3. Interleukin-1β

IL-1β is one of the first discovered cytokines and is significantly expressed by activated macrophages, dendritic cells, fibroblasts, endothelial cells, keratinocytes, and hepatocytes. Production of the active molecule requires inflammasome-mediated processing from caspases that cleave the inactive pro-IL-1β to yield the active cytokine that is then secreted. Immune cells can also secrete IL-1 receptor antagonist (IL-1ra), a soluble molecule that competes IL-1β for binding to the IL-1 receptor. However, IL-1ra binds to the IL-1 receptor non-productively thereby neutralizing the pro-inflammatory effect of IL-1β [107]. It is therefore the balance between IL-1β and IL-1ra levels that determines the magnitude of the inflammatory response. Importantly, recombinant human IL-1ra (anakinra) is used as treatment of rheumatoid arthritis and IL-1ra administration led to significant reduction of a panel of inflammation biomarkers in maintenance hemodialysis patients [108]. Accordingly, a study conducted in a big cohort of CKD patients demonstrated that plasma levels of several biomarkers of inflammation such as IL-1β, IL-1ra, IL-6, TNFα, as well as CRP and fibrinogen, were negatively associated with markers of kidney function and positively associated with albuminuria levels [109]. In an in vivo experimental setup, administration of an anti-IL-1β monoclonal antibody in LDL-receptor-deficient mice (Ldlr(−/−)) attenuated VC induced by a Western diet regimen, demonstrating the role of this cytokine in plaque formation [110]. Further, CPP deposits, a hallmark of VC processes, were shown to induce caspase-1 activation and IL-1β release from VSMCs providing an additional connection between these two mechanisms. Additional in vivo experiments reveal a close relation between IL-1β and VC. When CKD-induced rats were stimulated with a combination of calcium, phosphate and vitamin D they developed medial calcification of the thoracic aorta that coincided with high expression of IL-1β, IL-6, TNF, activation of NADPH oxidase and the concomitant suppression of several antioxidant enzymes [67]. In a similar fashion, human and murine VSMCs exposed to high glucose levels increased ALP activity and exhibited osteoblast trans-differentiation accompanied by IL-1β secretion, suggesting that glucose induces pro-calcifying VSMCs phenotype switch through IL-1β activation [111].

#### 3.4.4. Interleukin-8

Interleukin 8 (IL-8 or CXCL8), the first proinflammatory cytokine discovered in 1978, is secreted by many cell types but more typically by macrophages and microglia and after binding to its receptors CXCR1 and CXCR2. It functions as the main neutrophil chemoattractant in the bloodstream supporting migration of these cells into infected or injured tissues [112]. An increasing body of evidence supports the role of IL-8 in VC processes. Human umbilical vein endothelial cells exposed to inorganic phosphate and IS readily expressed and secreted IL-8 in the culture medium. Addition of this medium, in human aortic VSMC cultures, enhanced phosphate and IS induced calcification processes in a concentration-dependent manner. Moreover, as blockade of IL-8 or its receptors significantly reduced the pro-calcifying effect of phosphate and IS, IL-8 may be an important player in VC processes [113]. In line with this observation, clinical studies further support a correlation between IL-8 serum levels and VC incidence and extent [114,115] whilst IL-8 was identified as a prognostic marker of all-cause and cardiovascular mortality in hemodialysis patients [116].

#### 3.4.5. IL-18

Similar to IL-1β, IL-18 is produced as an inactive precursor that requires the catalytic action of inflammasome-derived caspases to produce the active molecule, hence the connection of inflammasome activation and inflammasome mediated cell death called pyroptosis with VC [117]. Initially described as an IFN-γ inducing factor as it facilitates T helper 1 (TH1) responses and natural killer (NK) cell activation, IL-18 is a member of the IL-1 family of cytokines and is mainly expressed by activated monocytes, macrophages, dendritic cells, Kupffer cells, keratinocytes, chondrocytes, synovial fibroblasts, and osteoblasts. Chondroblast and osteoblast production of IL-18 provides an initial link of this cytokine with calcification mechanisms. Among the targets of IL-18 are VSMCs that express the receptor for this cytokine and IL-18 signaling was found to induce the expression of several proinflammatory and pro-calcifying cytokines in VSMCs. This phenomenon was further enhanced by angiotensin II pretreatment of VSMCs [118]. Moreover, IL-18 was identified as a two-year follow-up predictor of cardiovascular death among non-diabetic CKD patients with a history of acute myocardial infarction in the previous year [119].

In addition to the aforementioned pro-inflammatory molecules, a recent review discussing the role of inflammatory cytokines as uremic toxins proposed a number of novel pro-inflammatory markers, such as IL-6R, IL-2, TNFR1, TNFR2, and chemokines, such as CXCL12 and CX3CL1, that should be added to the current list of uremic toxins due to their detrimental effects on the uremic phenotype [5].

### 3.5. Other Uremic Toxins

Leptin is an adipokine secreted principally by white adipose tissue and enterocytes, and its principal effect is to regulate energy homeostasis by decreasing appetite [120]. Leptin is classified as a uremic retention molecule exhibiting several pro-calcifying effects in in vitro human VSMC models [121], incrementing the proliferation of these cells, inducing the intracellular generation of ROS, and upregulating MMP-2 expression. Leptin also has been hypothesized to contribute to osteochondrogenic transition of VSMCs by stimulating TGFβ1 production that has been involved in these changes [122], and by inducing FGF23 overexpression [123]. The role of leptin in medial calcification has also been demonstrated in in vitro co-culture experimental models whereby VC was partially attributed to the secretion of leptin and vascular endothelial growth factor (VEGF) by adipocytes [124] and the direct induction of osteogenic differentiation of calcifying vascular cells [125]. Additional evidence from in vivo experiments support a role of leptin in osteoblast differentiation and pro-calcifying effects of VSMCs as leptin was able to downregulate the expression of the master mineralization inhibitor MGP whilst it was also able to induce the nuclear translocation of the main transcription factor β-catenin of the Wnt signaling pathway that mediates the VSMCs hyperplasia [126]. Furthermore, leptin treated murine models displayed an increment of osteogenic-specific markers like ALP, osteocalcin, and osteopontin [127]. Although several clinical studies have reported a strong association between serum leptin levels and VC in both CKD and elder populations [105,128,129,130,131,132], it was recently argued that because of insufficient evidence leptin should not be on the list of uremic toxins [5].

Indoxyl sulfate (IS) is a protein-bound uremic toxin derived from the metabolism of tryptophan predominantly bound to albumin. Although impaired renal clearance may be the main factor of IS accumulation [2,133], generation of uremic toxins in the gut is a major contributor to systemic levels, as demonstrated by absent or lower levels of uremic toxins in dialysis patients that have undergone colectomy [134]. As happens with other uremic toxins, higher levels of IS have been related to increased mortality [135] and CVD [136]. IS role in increased CVD seems to be multifactorial [137]. Different studies have demonstrated that IS induces endothelial disfunction, impairs VSMC regeneration and control, promotes a prothrombotic state and inhibits neoangiogenesis. The deleterious effects of IS accumulation on ECs are so diverse that it has lately been characterized as a uremic endotheliotoxin [138]. Different molecular pathways appear to mediate these detrimental effects. Briefly, IS appears to play a major role in promoting oxidative stress in ECs and VSMCs by inducing Nox4 and simultaneously reduce NO levels [139,140,141,142]. IS mediated ROS overproduction directly induces NF-kB and activating protein 1 (AP-1) nuclear translocation. Additionally, IS alters the expression profile of HIF-1α and VEGF in endothelial cell precursors therefore promoting their senescence and leading to reduced EC production and proliferation [143]. Moreover, in the presence of phosphate, IS induces secretion of the pro-inflammatory cytokine IL-8 [113]. Finally, IS induces VSMC proliferation and triggers the expression of transforming growth factor β (TGFβ), thereby promoting medial layer hyperplasia [144,145]. Therapeutic strategies aiming to decrease IS levels have gained relevance during the last years.

## 4. Possible Therapeutic Strategies

Considering the high mortality risk associated to VC in CKD patients, the search for novel strategies to prevent or decrease the burden of this disease is warranted. Up to date, no specific therapy has demonstrated strong direct clinical effects that effectively may be used to treat or prevent the progression of VC in CKD. Nevertheless, recent data from the ERA-EDTA registry revealed that absolute case-specific excess mortality due to atheromatous CVD present a decreasing trend from 2007 to 2015 in patients on renal replacement therapy [146].

**Figure 2:**
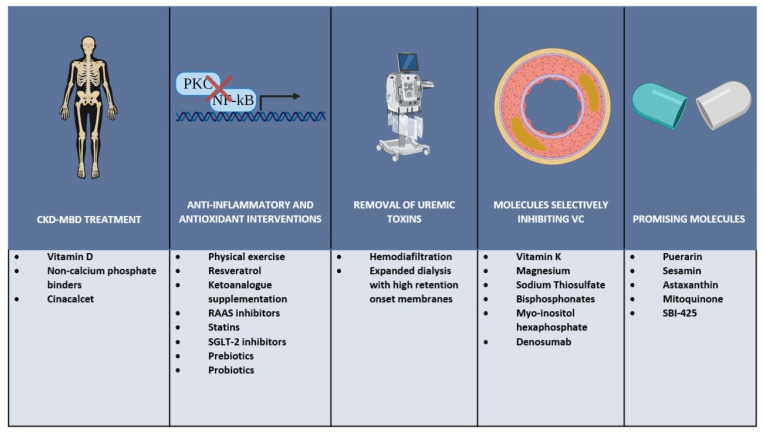
Possible therapeutic strategies for vascular calcification in CKD. Abbreviations: CKD-MBD: Chronic kidney disease-mineral and bone disorders; RAAS: Renin-angiotensin-aldosterone system; SGLT-2: Sodium-glucose cotransporter-2 SBI-425: 5-chloro-2-methoxyphenyl-sulfonamido-nicotinamide.

Together with the control of the biochemical components of chronic kidney disease mineral and bone disorders (CKD-MBD), some general strategies and commonly used drugs have shown, at least in experimental settings, beneficial effects in terms of controlling the factors leading to VC. Recently, new molecules have shown promising results in the prevention and treatment of VC and are now considered for clinical application pending further analysis in order to prove their safety and efficacy. Some of the possible interventions that could lead to a beneficial outcome in this group of patients are discussed below and summarized in Figure 2.

In order to prevent VC and delay its progression, a global approach is needed to concomitantly address many of the components involved in this complex disorder. Such an approach would need to include CKD-MBD treatment with special emphasis on the decrease of pro-calcifying factors like high phosphorus and calcium; anti-inflammatory and antioxidant interventions with lifestyle changes and administration of commonly used drugs; removal of uremic toxins by the use of appropriate renal replacement therapies especially of the large middle molecules accumulating in patients with ESKD; and the use of specific molecules with the potential of selectively inhibiting VC.

### 4.1. CKD-MBD Treatment

An essential part of the strategies to prevent VC in CKD is to control CKD-MBD. Different interventions have shown benefits in this regard, with the most important being the use of calcitriol, non-calcium-based phosphate binders and use of cinacalcet. As described earlier, hyperphosphatemia is associated with increased VC making phosphate binders an essential component in the management of patients with advanced CKD. However, the potential cardiovascular benefit of phosphate binders can be attenuated by the increase in calcium levels associated to the use of calcium-based binders. Accordingly, the use of non-calcium-based phosphate binders (e.g., sevelamer hydrochloride, sevelamer carbonate, and lanthanum carbonate) has demonstrated to be effective in controlling circulating phosphate levels. However, no clear benefits in terms of ameliorating VC and improving cardiovascular health could be proven [147]. On the other hand, cinacalcet modulates the activity of the calcium-sensing receptors in parathyroid tissue reducing serum concentrations of PTH and inducing upregulation of the calcium-sensing receptors in VSMCs and ECs. The use of cinacalcet has proven beneficial in lowering the serum levels of calcium, phosphate, and PTH in ESKD [148]. Additionally, a slower progression of VC was seen in CKD patients treated with cinacalcet and low doses of vitamin D [149,150].

### 4.2. Anti-Inflammatory and Antioxidant Interventions

Lifestyle changes have consistently shown a beneficial effect on inflammation and its associated complications. Physical exercise reduces inflammation and insulin resistance in CKD [151,152]. Aerobic exercise in hemodialysis patients improves endothelial function with enhanced flow-mediated vasodilation and reduced left ventricular hypertrophy [153]. Additionally, resistance exercise during dialysis has been found to increase Nrf2 expression and GPx activity [154]. Specific dietary interventions such as restriction of phosphorus intake represents since long cornerstones in the management of CKD-MBD. It is now increasingly recognized that healthy food with tailored diets including bioactive nutrients may have an important role to play to prevent and treat complications of CKD including VC [155] through influencing the disturbed uremic gut microbiota [156] or by other mechanisms. Among the many bioactive nutrients that could be supplied by food is resveratrol, a natural polyphenolic compound present in blueberries, grapes, and wine, with described antioxidant, anti-inflammatory, antiplatelet, and anticoagulant properties [157]. The use of ketoanalogue supplementation has also demonstrated beneficial effects on FGF-23 and Klotho that could be translated in CVD better outcomes [158]. Dietary Nrf2 agonists, such as sulforaphane, and/or dietary senolytics, such as quercetin and fisetin, may be alternative approaches to reduce cardiovascular risk in CKD [155].

In addition to lifestyle interventions, some commonly used drugs for treating different pathologies have shown a beneficial effect in reducing the production of uremic toxins or preventing their deleterious effects. Drugs targeting the renin-angiotensin-aldosterone system (RAAS) have an anti-inflammatory potential and were shown to reduce inflammatory markers in several small studies [159,160]. In this line, Olmesartan was found to exhibit potent protective effects against CKD-induced VC [161]. Statins represent another group of drugs commonly used among the general population due to their benefits in reducing mortality in patient with high cardiovascular risks. Apart from their lipid-lowering effect, statins display an anti-inflammatory potential, making them appealing candidates for preventing the progression of VC in CKD patients [162]. However, conflicting results have emerged since statin therapy was associated with greater progression of coronary artery calcification in ESKD [163].

Sodium-glucose cotransporter-2 (SGLT2) inhibitors have gained significant attention in the last years, not only because of their effect in controlling diabetes, but also because of their apparent cardioprotective effects. One SGLT2 inhibitor was recently found to reduce death from cardiovascular causes and prolong survival in CKD patients with or without type 2 diabetes, independently of the presence of CVD [164,165]. Among the described cardioprotective mechanisms [166], the reduction of inflammation appears especially relevant [167].

The altered uremic intestinal microbiome, results in an increased gut production of uremic toxins like IS, p-cresyl sulphate, and trimethylamine-N-oxide (TMAO) [168]. Several strategies have been tested in order to reduce intestinal uremic toxin production [155] for example through influencing the disturbed gut microbiota in CKD [156]. Although carbon oral absorbents slowed the progression of VC in animal models [169] this drug failed in human clinical studies [170]. Finally, the use of probiotics and prebiotics has shown a positive effect in the reduction of inflammatory cytokines in CKD [171], but it is uncertain if this translates into cardiovascular benefits.

### 4.3. Removal of Uremic Toxins

Newer dialysis techniques have been developed that increase the removal of uremic toxins. Online-hemodiafiltration reduces circulating levels of mediators of systemic inflammation and the expression of proinflammatory and prothrombotic microRNA particles, thus improving angiogenesis and reducing VSMC calcification [172]. Additionally, expanded dialysis (HDx) using medium cut-off membranes (MCO) which expand the limits of membrane permeability while providing increased selectivity for large solute removal has shown promising results [173]. This technique was shown to significantly reduce the gene expression of inflammatory cytokines (IL-6 and TNF) [174], and, moreover, reducing the pro-calcific potential of serum from dialysis patients in vitro compared to patients receiving high flux hemodialysis [175]. Further studies are warranted to demonstrate whether this positive effect also applies to in vivo VC.

### 4.4. Molecules Selectively Inhibiting Vascular Calcification

In the following, examples of molecules including pharmacological agents that were found to selectively inhibit VC processes in humans are described briefly.

#### 4.4.1. Vitamin K

Apart from its function as a cofactor of microsomal enzyme γ-glutamyl carboxylase (GGCX)-dependent hepatic clotting factor in coagulation, vitamin K is crucial in posttranslational protein modification by converting glutamic acid residues into γ-carboxyglutamate, i.e., vitamin K-dependent carboxylation [176,177]. The activation of vitamin K-dependent proteins (VKDPs) plays an important role in bone mineralization and ectopic VC. The role of vitamin K in VC has been well-established in preclinical studies, mainly through the carboxylation of VKDP MGP, whereby MGP knockout mice developed extensive calcification both in cartilage and vessels within 2 months [40]. Moreover, warfarin treatment (a vitamin K antagonist) induced coronary artery disease and vulnerable plaques in mice and vitamin K administration reversed such VC progression, suggesting the role of vitamin K in VC development [178]. However, the association between vitamin K status and VC in clinical observations are inconclusive [179,180,181] and the recent K4Kidneys trial failed to confirm the beneficial role of vitamin K supplement in vascular health [182].

#### 4.4.2. Magnesium

Experimental studies have demonstrated that magnesium inhibits VC. In an open label, randomized, controlled trial, enrolling patients with stage 3–4 CKD with risk factors for coronary artery calcification (CAC), magnesium supplementation resulted in slower progression of CAC compared to placebo. However, compliance of the treatment was limited due to adverse gastrointestinal effects [170]. Analyses of data of ongoing trials assessing the effects of magnesium supplementation in CKD-induced VC are pending [183].

#### 4.4.3. Sodium Thiosulfate

Sodium thiosulfate, an inorganic compound approved for the treatment of cyanide poisoning, has been used off-label to treat calciphylaxis in dialysis patients. The mechanism of action is thought to be through calcium chelation from precipitates in the skin, subcutaneous tissues, and organs, resulting in more soluble calcium salts, and it also seems to have antioxidant activity [184]. The use of sodium thiosulfate in dialysis patients delayed the progression of coronary, iliac artery, and cardiac valve calcification, as well as improved several cardiovascular risk factors like pulse wave velocity and left ventricular hypertrophy among others [185,186].

#### 4.4.4. Bisphosphonates

Bisphosphonates are antiresorptive drugs used for treating osteoporosis. First generation bisphosphonates, like etidronate, are pyrophosphate analogues, act as potent calcification inhibitors in bone and soft tissue [187]. Although these drugs are excreted by the kidney, therefore incrementing its bioavailability and half-life in patients with CKD, several studies have reassured its safety in CKD. The use of etidronate in hemodialysis patients suppressed the progression of CAC [188].

#### 4.4.5. Myo-Insitol Hexaphosphate

Promising data have been recently published regarding the results of the CaLIPSO trial, a phase 2b trial, where the effect of myo-inositol hexaphosphate (SNF472) on the progression of CAC in hemodialysis patients was examined [189]. SNF472 is an intravenous formulation of myo-inositol hexaphosphate, which inhibit the formation and progression of calcification in the soft tissue by binding to the growth sites of the hydroxyapatite crystal [190]. Since, SNF472 significantly attenuated the progression of coronary artery calcium and aortic valve calcification, with few mild adverse effects [189], it may be a promising novel therapeutic option.

#### 4.4.6. Denosumab

Denosumab is a human monoclonal antibody with high affinity and specificity for RANKL, approved for the treatment of osteoporosis and bone loss. Denosumab inhibits the binding of RANKL to its specific receptor RANK, thereby preventing activation, survival and further differentiation of osteoclasts and reducing bone resorption. In a single-center case series on VC on dialysis patients with secondary hyperparathyroidism, the effect of denosumab was studied. Results suggested that denosumab suppress the progression of CAC [191]. Limited data are available in the literature concerning the safety of denosumab in CKD. However, since monoclonal antibodies are not excreted via the kidneys, pharmacokinetic and pharmacodynamic properties are not modified in CKD, it seems safe in CKD [192]. Nonetheless, some reports have described the development of severe hypocalcemia in patients with CKD that received this medication [193].

### 4.5. Promising Novel Molecules

Additional molecules studied as potential inhibitors of VC have shown promising results both in in vitro and in vivo animal models. One example is puerarin, an isoflavonoid compound obtained from Chinese herbs with described anti-inflammatory, anti-oxidative and anti-apoptotic effects [194]. Liu et al. [195] demonstrated that puerarin administration attenuates VC by inhibiting inflammation. Sesamin, a lignin extracted from sesame seeds and sesame oil, has shown benefits in CVD [196]. Animal models exposed to this agent showed enhanced inhibition of platelet-derived growth factor (PDGF)-induced VSMC proliferation [197], attenuated degradation of collagen and elastin fibers [198] and diminished VSMC proliferation and migration [88]. Astaxanthin, is a carotenoid derivative found in the red pigments of crustacean shells and salmons with known potent antioxidative effect. High phosphate concentration treated VSMCs showed reduced pro-calcifying properties when exposed to astaxanthin [199]. Mitoquinone (a mitochondria-targeting antioxidant formed by covalently binding ubiquinone or coenzyme Q) attenuated VC by suppressing oxidative stress and apoptosis of VSMCs through the Keap1/Nrf2 pathway [200]. An inhibitor molecule of tissue-nonspecific ALP (TNAP), SBI-425 demonstrated efficacy in protecting against medial VC and improved survival in a CKD-MBD mouse model [201]. However, human studies for this inhibitor have not yet been carried out.

## 5. Conclusions

In summary, several uremic toxins accumulating in uremia play an important role in complex processes leading to VC and EVA (Figure 1). These processes are mediated by molecular mechanisms that induce EC and VSMC dysfunction, oxidative stress, activation of osteomorphogenesis mediating transcription factors, overexpression of extracellular matrix degrading enzymes, and deposition of HA crystals that provoke medial thickness. In addition to free water-soluble low molecular weight solutes that are removed by conventional dialysis, protein-bound solutes and large middle molecules including pro-inflammatory cytokines and chemokines represent an important threat. Their removal depends on advanced dialysis techniques using permeable membranes. While increasing the removal of uremic toxins by dialysis is important, effective preventive and therapeutic strategies addressing vascular calcification in CKD patients most likely will require also measures to reduce the formation of uremic toxins by intensified CKD-MBD treatment, anti-inflammatory, and antioxidant interventions. In addition, targeted nutritional interventions and pharmacological molecules that selectively inhibit VC might be added to our tools for combating EVA with calcification (Figure 2).

## Data Availability

Data sharing is not applicable to this article.
